# Medial Amygdala Lesions Selectively Block Aversive Pavlovian–Instrumental Transfer in Rats

**DOI:** 10.3389/fnbeh.2014.00329

**Published:** 2014-09-18

**Authors:** Margaret G. McCue, Joseph E. LeDoux, Christopher K. Cain

**Affiliations:** ^1^Emotional Brain Institute, Nathan Kline Institute for Psychiatric Research, Orangeburg, NY, USA; ^2^Center for Neural Science, New York University, New York, NY, USA; ^3^Child and Adolescent Psychiatry, New York University Medical School, New York, NY, USA

**Keywords:** medial, amygdala, Pavlovian, instrumental, transfer, avoidance, freezing, ultrasonic

## Abstract

Pavlovian conditioned stimuli (CSs) play an important role in the reinforcement and motivation of instrumental active avoidance (AA). Conditioned threats can also invigorate ongoing AA responding [aversive Pavlovian–instrumental transfer (PIT)]. The neural circuits mediating AA are poorly understood, although lesion studies suggest that lateral, basal, and central amygdala nuclei, as well as infralimbic prefrontal cortex, make key, and sometimes opposing, contributions. We recently completed an extensive analysis of brain c-Fos expression in good vs. poor avoiders following an AA test (Martinez et al., 2013, *Learning and Memory*). This analysis identified medial amygdala (MeA) as a potentially important region for Pavlovian motivation of instrumental actions. MeA is known to mediate defensive responding to innate threats as well as social behaviors, but its role in mediating aversive Pavlovian–instrumental interactions is unknown. We evaluated the effect of MeA lesions on Pavlovian conditioning, Sidman two-way AA conditioning (shuttling) and aversive PIT in rats. Mild footshocks served as the unconditioned stimulus in all conditioning phases. MeA lesions had no effect on AA but blocked the expression of aversive PIT and 22 kHz ultrasonic vocalizations in the AA context. Interestingly, MeA lesions failed to affect Pavlovian freezing to discrete threats but reduced freezing to contextual threats when assessed outside of the AA chamber. These findings differentiate MeA from lateral and central amygdala, as lesions of these nuclei disrupt Pavlovian freezing and aversive PIT, but have opposite effects on AA performance. Taken together, these results suggest that MeA plays a selective role in the motivation of instrumental avoidance by general or uncertain Pavlovian threats.

## Introduction

Instrumental active avoidance (AA) is a major mechanism for coping with threats. As with all forms of defensive conditioning, AA mechanisms evolved because they were adaptive. Indeed, AA gives subjects control in dangerous situations and likely contributes to adaptive active coping strategies and resilience (LeDoux and Gorman, 2001). However, when active avoidance responses (ARs) are inappropriate, or occur too frequently, they can interfere with normal activities and contribute to anxiety pathology (McGuire et al., 2012). Compared to related phenomena like Pavlovian threat conditioning (Johansen et al., 2011), very little is known about the brain mechanisms of AA.

In a typical signaled AA paradigm, rats first learn that a conditioned stimulus (CS, sometimes called a “warning signal”; e.g., tone) predicts the occurrence of an aversive unconditioned stimulus (US; e.g., footshock). This Pavlovian phase transforms the CS into a threat that triggers defensive reactions (e.g., freezing). Then, on subsequent trials, rats gradually learn to suppress Pavlovian reactions and emit a specific instrumental action (AR; e.g., shuttle) that terminates the CS and prevents US delivery. Although the reinforcement mechanism in AA is unknown, one prominent theory hypothesizes that “fear reduction” associated with CS termination reinforces the AR (Mowrer and Lamoreaux, 1946; Miller, 1948; Rescorla and Solomon, 1967; Levis, 1989). Conditioned threats also play an important role in AA expression; once the instrumental contingency is acquired, CS presentations provide the motivation to perform the AR (Rescorla, 1990).

Active avoidance learning is also possible without an explicit CS or warning signal (Sidman, 1953). In the unsignaled AA paradigm, rats learn to emit ARs at regular intervals to delay US presentations (Bolles and Popp, 1964). In this task, similar Pavlovian and instrumental processes are hypothesized; however, the CS is a contextual cue that increases in intensity with time (Anger, 1963; Rescorla, 1968). Although unsignaled AA is more difficult to learn than signaled AA, it has proven useful for addressing some key questions about AA mechanisms. For instance, we have exploited the variability in unsignaled AA behavior to demonstrate that AA performance reflects a competition between competing motivations to react (e.g., Pavlovian freezing) or act (e.g., instrumental shuttle) in the face of threat (Lazaro-Munoz et al., 2010). Further, since unsignaled AA produces a steady rate of ARs, it is ideal for studying aversive conditioned motivation mechanisms in isolation with Pavlovian–instrumental transfer tasks (PIT; Rescorla and Lolordo, 1965; Patterson and Overmier, 1981; Laroche et al., 1987; Campese et al., 2013). In the aversive PIT procedure, Pavlovian and instrumental conditioning occur separately. Then, during the critical PIT test, AR rates are compared during CS and CS-free periods. Aversive CSs facilitate AA responding, most likely by activating a central arousal-like state (LeDoux, 2014).

We have used signaled AA, unsignaled AA, and aversive PIT tasks to help reveal the neural circuitry of AA and to identify areas that may contribute to conditioned motivation or response competition. In a recent study, we evaluated expression of the immediate-early gene *c-fos* after unsignaled AA training (Martinez et al., 2013). Good avoiders showed high AR rates and low freezing, whereas poor avoiders showed an opposite pattern. Although we examined a number of brain regions, we found that c-Fos expression correlated with freezing and AA behavior in only five regions: lateral amygdala (LA), basal amygdala (BA), central amygdala (CeA), infralimbic prefrontal cortex (IL-PFC), and medial amygdala (MeA). Involvement of the first four regions in AA converges with lesion studies. Lesions of LA or BA block AA acquisition and impair AA expression (Poremba and Gabriel, 1997, 1999; Choi et al., 2010; Lazaro-Munoz et al., 2010). Lesions of CeA block Pavlovian freezing and facilitate AA in poor avoiders, but have little effect in good avoiders (Choi et al., 2010; Lazaro-Munoz et al., 2010; Moscarello and LeDoux, 2013). Lesions of IL-PFC enhance freezing and impair AA acquisition (Moscarello and LeDoux, 2013). And lesions of LA or CeA, but not BA, impair aversive PIT (Campese et al., 2014). Considered with findings from Pavlovian conditioning studies (reviewed by Cain and LeDoux, 2008a), this has led us to a hypothetical model where LA is critical for learning and storing Pavlovian CS–US associations, and this information can be used in different ways to: (1) elicit Pavlovian reactions (via CeA), (2) motivate specific instrumental actions linked to the CS (via BA), or (3) generally motivate instrumental actions (via CeA). IL-PFC contributes by suppressing CeA-mediated reactions that compete with ARs. This is an incomplete working model as much remains unknown; however, these studies begin to address how the neural circuits of Pavlovian and instrumental aversive conditioning interact to produce behavior in the AA paradigm.

The only brain region identified by our c-Fos analysis that has not been investigated with lesions in the AA task is MeA. MeA is part of the extended amygdala (Alheid et al., 1995), a collection of structures that have been generally implicated in risk assessment and low-level defensive behaviors to uncertain or distant threats (Kemble et al., 1984; Davis et al., 2010). MeA has also been clearly implicated in innate defensive responses to predator cues (Rosen et al., 2008; Takahashi et al., 2008), as well as aggression and sexual behavior (Newman, 1999). MeA disruption has been studied with Pavlovian conditioning, although the results have been mixed (Nader et al., 2001; Walker et al., 2005). To our knowledge, the effects of MeA lesions on AA or aversive PIT have never been evaluated. MeA receives projections from LA and CeA and could mediate CS-elicited reactions that compete with ARs (Pitkänen, 2000). MeA also receives inputs from IL-PFC and could be necessary for suppressing CeA-mediated reactions that compete with ARs (McDonald et al., 1999). Finally, MeA projects to regions like the ventral tegmental area and striatum that may be important for instrumental learning and conditioned motivation to act (Pardo-Bellver et al., 2012).

Given this sparse information, we tentatively hypothesized that MeA is required for Pavlovian motivation of AA performance, but not for Pavlovian defensive reactions. To test this, we used electrolytic lesions of MeA and evaluated unsignaled AA, Pavlovian conditioning, and aversive PIT behaviors. Pre- and post-training lesions were used to differentiate between effects on learning and performance in the AA task. Further, we designed the studies to measure a range of defensive reactions to learned and innate threat stimuli in order to clarify the role of MeA in defensive conditioning. Finally, we included several control measures to determine whether MeA lesions affect basic sensorimotor functions. The results suggest that MeA selectively mediates low-level defensive reactions and motivation of instrumental avoidance by general or uncertain threats.

## Materials and Methods

### Subjects

Subjects were 74 Male Sprague-Dawley rats (Hilltop Lab Animals Inc., Scottsdale, PA, USA) weighing ~300 g at the start of the study. Rats were housed two per cage and maintained on a 12:12-h light:dark schedule with free access to food and water. All experiments were approved by the Nathan Kline Institute Animal Care and Use Committee and were in accordance with NIH guidelines.

### Apparatus

All avoidance, avoidance extinction, and PIT sessions occurred in standard rat two-way shuttleboxes (H10-11R-SC; Coulbourn Instruments, Whitehall, PA, USA). Shuttleboxes were equipped with infrared beam arrays to automatically detect movement between chamber sides, and bat detectors for analysis of 22 kHz ultrasonic vocalizations (USVs; Noldus Ultravox system, Leesburg, VA, USA). Pavlovian threat conditioning and context freezing tests occurred in standard rat conditioning boxes (H10-11R-TC; Coulbourn Instruments). Shuttleboxes and conditioning boxes also contained house lights, infrared indicator lights, video cameras, 8 ohm speakers (one per conditioning box, two per shuttlebox on opposite ends) and stainless steel grid floors for scrambled footshock delivery (shock source: Precision Animal Shocker, model H13–15, Coulbourn Instruments). Tone stimuli were delivered to speakers by programmable tone generators (Coulbourn Instruments, model A12–33). Shuttleboxes and conditioning chambers were enclosed in sound attenuating chambers (H10-24A). All conditioning procedures were controlled by Graphic State software (v3.03, Coulbourn Instruments). Predator odor tests occurred in a custom two-compartment chamber with wire mesh floors. Each chamber measured 28 cm × 28 cm × 43 cm (L × W × H) and was open at the top to allow for recording of animal behavior via an overhead video camera. The internal walls were painted gray and chambers sides were indistinguishable. Chamber sides were separated by a small open passage (10 cm × 19 cm). Pavlovian cue freezing tests occurred in Coulbourn conditioning boxes modified to mask salient contextual cues. Modifications included: plastic inserts to cover grid floors, high contrast visual cues added to transparent walls, and the addition of a novel odor (floor pans cleaned with 6% ethanol before test). Key behavioral sessions were recorded to DVD for offline analyses.

### Procedure

**Figure 1:**
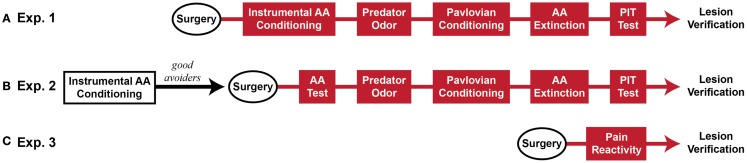
**Experimental timeline (~2 months beginning to end)**. **(A)** Rats received Sham or MeA-lesion surgery prior to all behavioral testing. **(B)** Rats received unsignaled Sidman AA training for seven daily sessions. Poor avoiders were identified after session 7 and excluded from further study. Good avoiders received Sham or MeA-lesion surgery followed by behavioral testing. **(C)** In a final, separate experiment, rats received Sham or MeA-lesion surgery followed by pain reactivity testing only. Red behavioral stages all occurred post-lesion.

Five sequential behavioral phases comprised the major experiments: (1) Sidman active avoidance conditioning, (2) predator odor tests, (3) Pavlovian threat conditioning, (4) avoidance extinction, and (5) Pavlovian–instrumental transfer tests. Two experiments were conducted, which differed mainly in the timing of MeA lesions. In Experiment 1, MeA lesions occurred before all behavioral phases. In Experiment 2, MeA lesions occurred after avoidance training and before all other behavioral phases. Additionally, cat hair served as the predator odor in Experiment 1 and fox urine served as the predator odor in Experiment 2. Finally, in Experiment 2, poor avoiders were identified after AA training and excluded from further analysis, thus, MeA lesions were evaluated only in good avoiders. After these experiments were completed, a third experiment was conducted to determine if MeA lesions affect pain threshold. At the completion of behavioral testing, rats were transcardially perfused under deep anesthesia and brains were removed for histological verification of lesions. See Figure 1 for experimental timelines.

#### Unsignaled AA conditioning

Rats received unsignaled Sidman AA training (4–5 sessions per week, 1 session per day, 25 min per session) as previously described (Lazaro-Munoz et al., 2010; Campese et al., 2013, 2014) where every shuttling response (movement to the opposite chamber side) delayed the delivery of the unconditioned stimulus (US; 0.5 s × 1 mA footshock) by 30 s (R–S or response–shock interval). In the absence of shuttling, the US was delivered every 5 s (S–S or shock–shock interval). Avoidance responses (ARs) were defined as shuttles during the R–S interval; shuttles during the S–S interval were considered escape responses (ERs). All shuttles were marked by a brief feedback stimulus (house lights blink off for 0.3 s). The number of ARs, ERs, shocks, and USVs were automatically recorded for all sessions. In Experiment 1, all rats received eight post-lesion training sessions. In Experiment 2, rats received seven training sessions, then surgery and recovery, followed by two additional AA sessions (identical to training). Note that poor avoiders were identified after session seven and excluded from further analysis as previously described (Lazaro-Munoz et al., 2010).

#### Predator odor

Predator odor tests were included as a positive control for effective MeA lesions (reviewed by Takahashi et al., 2005). Rats received two habituation sessions and two predator odor test sessions on consecutive days. Each session lasted 10 min and the percentage of total time spent in each chamber side was measured from video files. In Experiment 1, cat hair and a segment of cat collar were placed in a receptacle under the wire mesh floor in one compartment of the chamber (Blanchard et al., 2005). Since no effect of cat odor was found, in Experiment 2, 50 μl of 100% fox urine (Leg Up Enterprises, Lovell, ME, USA) was pipetted onto a Kimwipe and placed in a receptacle under the wire mesh floor of one compartment. No attempt was made to actively control odor flow between the compartments. To evaluate the effect of MeA lesions on predator odor, both the habituation sessions and predator odor tests were analyzed from video files to quantify the time spent in each chamber. To evaluate potential effects of MeA lesions on locomotor activity, habituation sessions were analyzed from video files by bisecting each chamber into quadrants with lines on the video monitor and counting the number of line crossings during the session.

#### Pavlovian threat conditioning

Rats received three pairings of the conditioned stimulus (CS: 30 s, 5 kHz, 80 dB tone) and co-terminating US (0.7 mA × 1 s footshock) with 3 min acclimation and inter-trial intervals. One day later, rats received counterbalanced cue and context tests separated by 3 h. For the context test, rats were returned to the conditioning boxes for 8 min. For the cue test, conditioning chambers were modified to remove salient contextual cues, and a 30-s CS was presented 3 min after entry to the chamber. Freezing was rated from DVD files by an experienced observer blind to treatment condition. For cue tests, freezing was rated continuously and percent freezing was calculated by dividing the total seconds freezing by 30 and multiplying by 100. For context tests (including freezing during AA training), freezing was rated by time-sampling; every 5 s the rater determined whether the rat was freezing or not, and percent freezing was calculated by dividing the number of freezing observations by the total number of observations and multiplying by 100.

#### AA extinction

Rats were returned to the shuttleboxes for 60 min with shockers turned off. Feedback was provided with each shuttle response. Long-term memory for AA extinction was assessed during the first 5 min of the PIT test session, 1 day after extinction training.

#### Pavlovian–instrumental transfer

Rats received two PIT tests separated by 1 day. PIT test sessions involved a single presentation of the aversive CS in the shuttleboxes while rats shuttled under extinction (US presentations absent, response feedback present). For each individual, the CS presentation was triggered when the shuttling rate fell below two responses per minute (RPMs) for two full minutes. Previous work found that PIT effects were greatest when baseline response rates were low (~2 RPMs), but not absent (Campese et al., 2013). Since rats vary greatly in their rates of AA extinction, this protocol ensured similar baseline response rates when PIT was assessed. Additionally, since some rats freeze when initially placed in the shuttleboxes, the CS trigger was disabled for the first 15 min of USAA extinction. Once triggered, the CS presentation remained on until 10 shuttles were performed. Immediately after the 10th shuttle response, the CS was terminated, the house light turned off and the session ended. For each rat in each test, a PIT score was calculated by the following equation: (shuttling rate during the CS/shuttling rate during an equivalent Pre-CS period)*100.

#### Shock reactivity

Rats were placed individually into the conditioning boxes and scrambled 0.5 s footshocks were delivered every 30 s, beginning 60 s after entry to the chamber. The initial shock intensity was 0.1 mA and each subsequent shock increased by 0.1 mA. Thresholds to flinch, vocalize, and jump were recorded as described by others (Swedberg, 1994). The session was terminated once a jump was observed or 1.5 mA was reached; however, all rats emitted jump responses prior to reaching the 1.5-mA maximum.

### Surgery

Rats were anesthetized with isoflourane (3–4%) (Henry Schein, Melville, NY, USA), and placed in a stereotaxic apparatus (David Kopf Instruments, Tujunga, CA, USA). Small burr holes were drilled in the skull above MeA. A stainless steel monopolar electrode covered with epoxy (exposed tip of 500 μm; model NE-300X, David Kopf Instruments) was lowered through an incision in the dura into MeA. Bilateral lesions were created with a lesion maker (model 53500, Ugo Basile, Italy) by passing current (+ 0.5 mA, 12 s) through the electrode at four different drop sites (relative to Bregma in millimeters): (1) AP: −1.9, ML: ± 3.2, DV: −9.2; (2) AP: −2.4, ML: ± 3.2, DV: −9.3; (3) AP: −2.9, ML: ± 3.4, DV: −9.0; (4) AP: −3.4, ML: ± 3.4, DV: −8.9. Post-operative pain was managed with subcutaneous Buprenorphine SR (0.5 mg/kg; ZooPharm, Windsor, CO, USA). Sham animals underwent the same procedure, but no current was passed through the electrode. Animals recovered in their homecages, singly housed, for 14 days following surgery, and then were returned to pair housing for the remainder of the experiment.

### Lesion verification

At the completion of behavioral testing, rats were given an anesthetic overdose and perfused transcardially with 10% phosphate-buffered formalin. Brains were removed and stored in 10% phosphate-buffered formalin and 30% sucrose for at least 3 days and were then cut in 50 μm sections using a freezing microtome (every other section was collected). Nissl stains were then performed and tissue images were collected (Nikon Microphot-FXA). Damage to target brain regions and adjacent areas was assessed using a rat brain atlas as a guide (Paxinos and Watson, 2005).

### Statistical analysis

Data are presented as group means (±SEM). Bar graphs with two groups were analyzed with unpaired, two-tailed student’s *t*-tests. All other data were analyzed with two-way repeated measures ANOVAs (GraphPad Prism 6.0, GraphPad Software Inc., La Jolla, CA, USA). Planned *post hoc* comparisons were analyzed using Bonferroni’s Multiple Comparison test. Differences were considered significant if *p*-values were less than 0.05. Note that behavioral results from Experiments 1 and 2 were initially analyzed separately. Data from Experiments 1 and 2 were combined only if: (1) testing occurred post-lesion in both experiments, (2) MeA lesions produced the same outcome (effect vs. non-effect) in both experiments, and (3) direct comparisons revealed no statistically significant differences between Sham groups or MeA-lesion groups from each experiment.

## Results

Avoidance data were analyzed separately for Experiments 1 and 2, since MeA lesions occurred pre- or post-training. With the exception of the predator odor data, all other tests combined data from Experiments 1 and 2 for analysis since these tests all occurred post-lesion and there were no differences in MeA lesion effects between the experiments. The predator odor tests were also analyzed separately since they used different odors (cat hair vs. fox urine).

### Lesion verification

**Figure 2:**
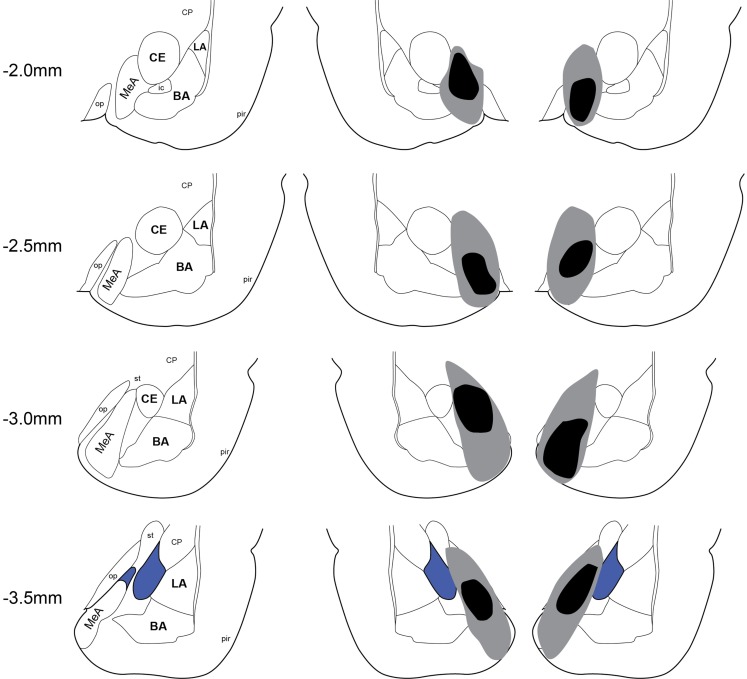
**Lesion placements**. Shaded areas represent the greatest (gray) and least (black) extent of electrolytic lesions. Numbers on the left represent distance from Bregma in millimeters. Brain slides adapted from Paxinos and Watson (2005) with permission from Elsevier. LA, lateral amygdala; BA, basal amygdala; CeA, central amygdala; MeA, medial amygdala; pir, piriform; op, optic tract; CP, caudate putamen.

Twenty-two rats received Sham lesion surgery and 42 rats received electrolytic lesions targeted to MeA. Twelve rats died post-surgery and all of these were in the MeA lesion group. Figure 2 depicts the extent of acceptable lesions to MeA in the final dataset. One rat was excluded because of insufficient bilateral damage to MeA or excessive damage to adjacent regions. Thus, the final groups included 22 shams (Experiment 1: *n* = 9; Experiment 2: *n* = 5; Experiment 3: *n* = 8) and 27 MeA lesions (Experiment 1: *n* = 13; Experiment 2: *n* = 8; Experiment 3: *n* = 6).

### Active avoidance measures

**Figure 3:**
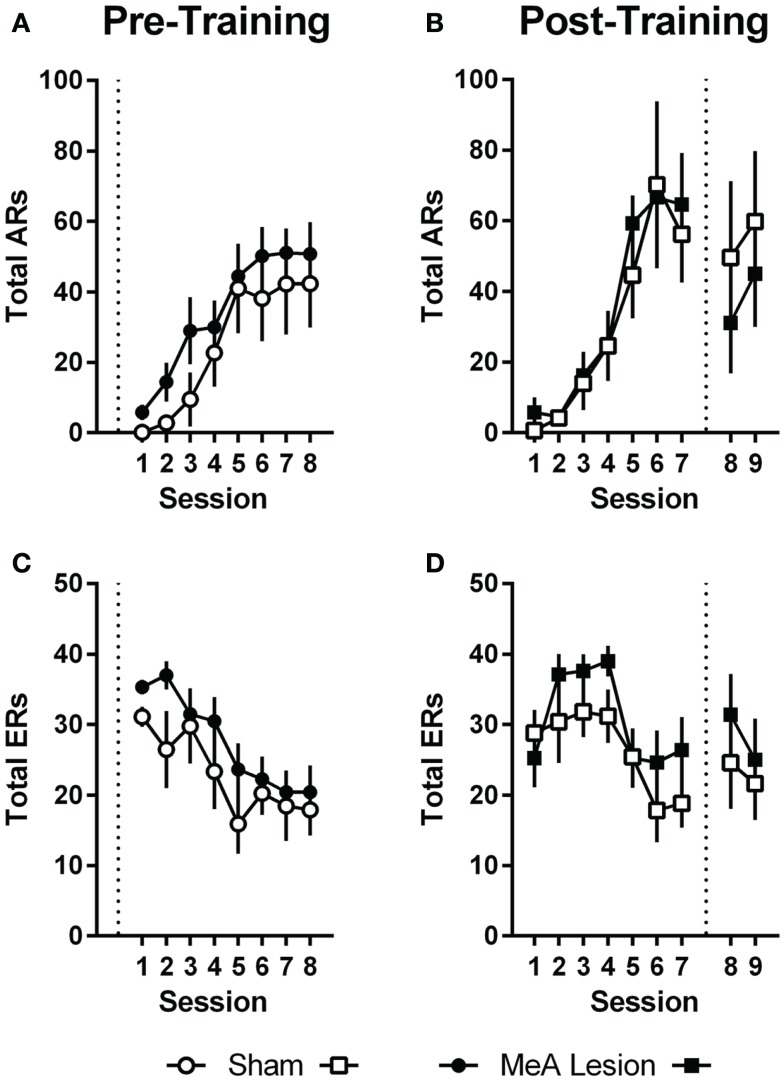
**MeA lesions have no effect on AA learning or performance**. **(A,C)** AA responses (ARs) and escape responses (ERs) during AA training for rats with pre-training MeA (filled circles, *n* = 13) or sham (open circles; *n* = 9) lesions. **(B,D)** ARs and ERs during AA training (sessions 1–7) and AA tests (sessions 8–9) for rats with post-training MeA (filled squares; *n* = 8) or sham (open squares; *n* = 5) lesions. Dotted vertical lines represent lesion surgeries in relation to AA training and testing.

Pre-training lesion effects on AA measures were analyzed using group (Sham vs. lesion) × session (1–8) ANOVAs. Session was treated as a repeated measure. Bonferonni post-tests evaluated group effects for individual sessions. Rats in both groups acquired the AA task equally (Figure 3); AA responses increased with training [group: *F*_(1,20)_ = 1.0, *p* = 0.32; session: *F*_(7,140)_ = 17.2, *p* < 0.01; group × session: *F*_(7,140)_ = 0.32, *p* = 0.94] and escape responses (ERs) decreased with training [group: *F*_(1,20)_ = 1.6, *p* = 0.22; session: *F*_(7,140)_ = 9.3, *p* < 0.01; group × session: *F*_(7,140)_ = 0.71, *p* = 0.66]. Rats in both groups also saw a decline in the number of shocks as the AR was acquired, although MeA-lesion rats received fewer shocks throughout training [group: *F*_(1,20)_ = 6.4, *p* = 0.02; session: *F*_(7,140)_ = 14.8, *p* < 0.01; group × session: *F*_(7,140)_ = 0.85, *p* = 0.54; Figure S1A in Supplementary Material]. To better understand how MeA-lesion rats could experience fewer shocks than Sham rats, but exhibit similar numbers of ARs and ERs, we more closely evaluated the patterns of responding during session 1 of training. Interestingly, MeA-lesion rats were more likely to escape following a shock presentation (Figure S1A in Supplementary Material). Since shocks are delivered every 5 s in the absence of shuttling, it is possible to receive significantly fewer shocks while still performing similar numbers of AR and ER shuttles. Note that MeA-lesion rats performed slightly more ARs and ERs in each session of training, thought this difference was statistically insignificant.

For Experiment 2, pre-lesion AA data was analyzed as above, with repeated measures group (Sham vs. lesion) × session (1–7) analyses. To evaluate the effect of lesions on AA in good avoiders, we compared the average of the final two AA training sessions (6–7) to the average of the two post-lesion test sessions (8–9) with group (Sham vs. lesion) by phase (pre- vs. post-lesion) ANOVAs, treating Phase as a repeated measure. Poor avoiders, identified after session 7, were excluded from all analyses. Rats in both groups acquired AA equally prior to lesion surgeries; there were no differences in ARs, ERs or shocks [group effects: *F*_(1,11)_ < 1.9, *p* > 0.19; session effects: *F*_(6,66)_ > 5.0, *p* < 0.01; group × session effects: *F*_(6,66)_ < 0.77, *p* > 0.59]. Although there was a slight dropoff in ARs following the lesion and recovery period for both groups [Phase: *F*(_1,11)_ = 0.01, *p* = 0.01], MeA lesions had no effect on ARs, ERs, or shocks post-lesion [group × phase interactions: *F*_(1,11)_ < 2.6, *p* > 0.14].

### 22 kHz USVs and freezing during AA

**Figure 4:**
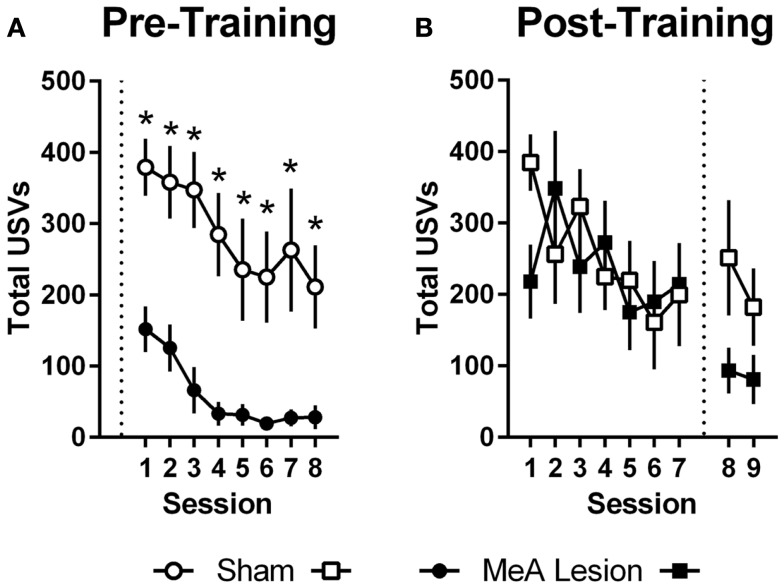
**MeA lesions impair USVs in the AA context**. **(A)** Total number of 22 kHz USVs per session during AA training for rats with pre-training MeA (filled circles; *n* = 13) or sham (open circles; *n* = 9) lesions. **(B)** Total USVs during AA training (sessions 1–7) and AA tests (sessions 8–9) for rats with post-training MeA (filled squares; *n* = 8) or sham (open squares; *n* = 5) lesions. *p < 0.05 vs. Sham controls.

Ultrasonic vocalizations were automatically recorded throughout AA training and testing in the shuttleboxes. We also rated freezing behavior during the first 2 min of each training and test session. Statistical analyses of USV and freezing data for Experiments 1 and 2 were identical to those described for AA measures above (see Active Avoidance Measures). In Experiment 1, USVs declined as ARs were acquired, and rats with pre-training MeA lesions showed profound impairments in USVs throughout AA training [Figure 4A; group: *F*_(1,20)_ = 24.00, *p* < 0.01; session: *F*_(7,140)_ = 9.20, *p* < 0.01; group × session: *F*_(7,140)_ = 0.68, *p* = 0.69]. In Experiment 2, USVs also declined as ARs were acquired, and there were no differences between the groups prior to lesions [Figure 4B; group: *F*_(1,11)_ = 0.06, *p* = 0.81; session: *F*_(6,66)_ = 2.32, *p* = 0.04; group × session: *F*_(6,66)_ = 1.67, *p* = 0.14]. Due to equipment failure, USV data were lost during the post-lesion test for three rats (two Sham and one MeA-lesion rat). Rats with MeA lesions showed a decline in USVs compared to Shams; however, the differences were not statistically significant [group: *F*_(1,8)_ = 1.8, *p* = 0.21, phase: *F*_(1,8)_ = 5.0, *p* = 0.06, group × phase: *F*_(1,8)_ = 1.3, *p* = 0.29].

**Figure 5:**
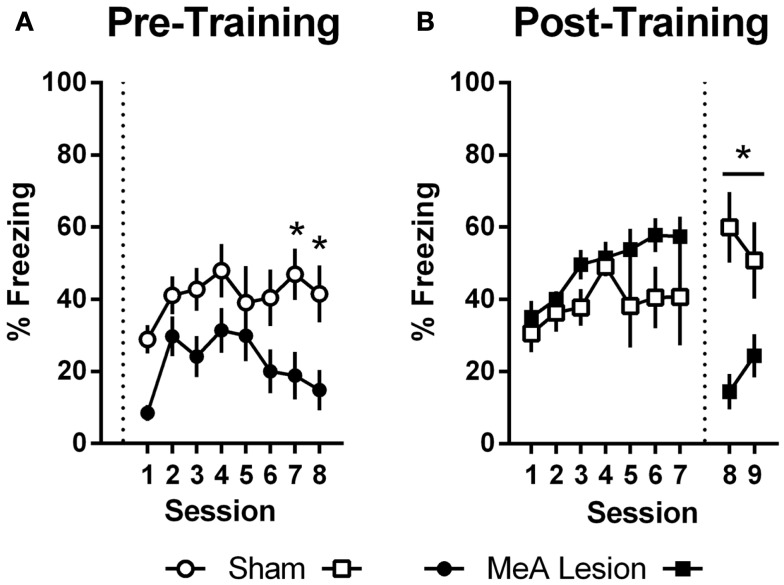
**MeA lesions impair freezing in the AA context**. **(A)** Percent time spent freezing during the first 2 min of each training session for rats with pre-training MeA (filled circles; *n* = 13) or sham (open circles; *n* = 9) lesions. **(B)** Percent freezing during the first 2 min of AA training (sessions 1–7) and AA tests (sessions 8–9) for rats with post-training MeA (filled squares; *n* = 8) or sham (open squares; *n* = 5) lesions. *p < 0.05 vs. Sham controls.

In Experiment 1, freezing in the shuttleboxes increased with AA training and MeA-lesion rats froze significantly less than Sham controls [Figure 5A; group: *F*_(1,20)_ = 6.55, *p* = 0.02; session: *F*_(7,140)_ = 4.65, *p* < 0.01; group × session: *F*_(7,140)_ = 1.30, *p* = 0.26]. Bonferonni post-tests indicate that the strongest group differences were toward the end of AA training [sessions 7 and 8: *t*_(160)_ > 1.9, *p* < 0.05]. In Experiment 2, freezing also increased with AA training, and there were no differences between the groups prior to lesions [Figure 5B; group: *F*_(1,11)_ = 3.09, *p* = 0.11; session: *F*_(6,66)_ = 3.56, *p* < 0.01; group × session: *F*_(6,66)_ = 0.90, *p* = 0.50]. However, after surgery, Sham rats showed slightly increased freezing rates in the AA context whereas MeA-lesion rats showed decreased freezing [group × phase: *F*_(1,11)_ = 43.71, *p* < 0.01]. Bonferonni post-tests confirmed the absence of a group difference pre-lesion [*t*_(22)_ = 1.83] and a significant group difference post-lesion [*t*_(22)_ = 3.88, *p* < 0.01].

Since rats with pre-training MeA lesions received fewer shocks than Sham controls in Experiment 1 (Figure S1A in Supplementary Material), we conducted an additional analysis to determine if this explained the reduction in USVs and freezing in the shuttleboxes during AA training. Sham and MeA-lesion groups were divided in half by the number of shocks received during session 1 of AA training. Rats in the bottom half of the Sham group (Sham-Low; *n* = 5) and those in the top half of the MeA-lesion group (MeA-High; *n* = 6) had nearly identical mean shock scores (114 vs. 115). We compared Shocks, ARs, ERs, USVs, and freezing with two-way group (Sham-Low vs. MeA-high) × session (1–8) ANOVAs, treating session as a repeated measure. We found no differences in Shocks, ARs, ERs, or freezing [group effects: *F*_(1,91)_ ≤ 1.41, *p* > 0.27]. However, MeA-lesion rats still showed significantly fewer USVs even after controlling for shock levels [Figure S2 in Supplementary Material; group: *F*_(1,9)_ = 32.60, *p* < 0.01; session: *F*_(7,63)_ = 14.57, *p* < 0.01; group × session: *F*_(7,63)_ = 2.56, *p* = 0.02].

### Predator odor

As a positive control for MeA lesions, we also tested avoidance of predator odors, by comparing the percent time spent in the predator odor chamber during two habituation sessions (no predator odor) and two test sessions (predator odor in one side). For the statistical analysis, we took the average of the habituation and test sessions and conducted two-way group (Sham vs. lesion) × phase (habituation vs. odor test) ANOVAs, treating phase as a repeated measure. In Experiment 1, we found no effect of cat hair odor on the time spent in the cat hair chamber, thus, it was impossible to evaluate the effects of MeA lesions on cat hair avoidance [Phase: *F*_(1, 20)_ = 1.4, *p* = 0.26]. Thus, in Experiment 2, we switched to fox urine as our predator odor, as this may be a more salient natural threat cue (Takahashi et al., 2005; Fendt, 2006). In this experiment, Sham rats showed a reduction in time spent in the fox urine chamber, and MeA-lesion rats did not [Figure S3 in Supplementary Material; group × phase interaction: *F*_(1,11)_ = 6.7, *p* = 0.03]. Bonferonni post-tests confirmed that there were no differences between the groups during habituation [*t*_(22)_ = 0.06], but MeA-lesion rats spent more time in the fox urine chamber during the test [*t*_(22)_ = 2.52, *p* < 0.05]. Interestingly, MeA-lesion rats appeared to prefer the fox urine chamber, perhaps because they experience the odor as less aversive than shams and are more likely to investigate this novel stimulus.

### Locomotor activity

To evaluate potential MeA lesion effects on baseline locomotor activity, we measured line crossings during the predator odor habituation sessions. For each animal, an average of the two sessions was calculated. There were no differences in locomotor activity for the groups in Experiments 1 and 2, so these were combined into a single analysis. We found no differences in locomotor activity between Sham and MeA-lesion rats [Figure S4A in Supplementary Material; *t*_(33)_ = 1.08, *p* = 0.29].

### Pavlovian threat conditioning

**Figure 6:**
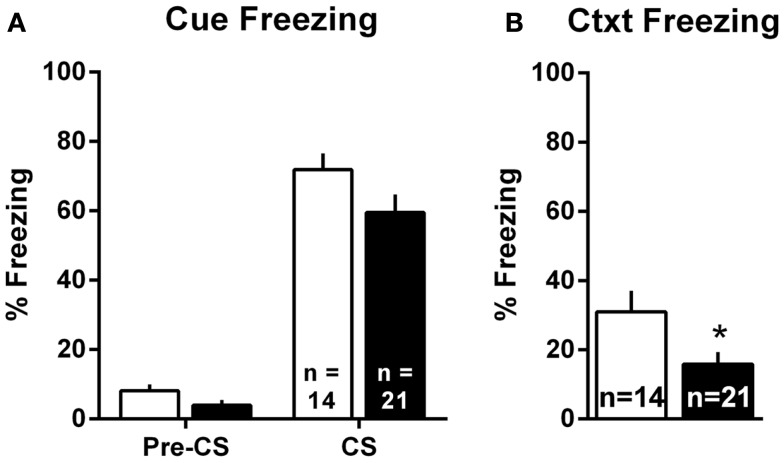
**MeA lesions impair context, but not cue, freezing outside the AA context**. Rats received auditory Pavlovian threat conditioning in a novel context followed 1 day later by counterbalanced tests of cue and context freezing. **(A)** Percent time spent freezing during the 30-s pre-CS and CS periods for all MeA (filled bars, *n* = 21) and sham (open bars; *n* = 14) rats in Experiments 1 and 2. **(B)** Percent time spent freezing during the 8-min exposure to the Pavlovian conditioning context for same rats. *p < 0.05 vs. Sham controls.

Pavlovian threat conditioning occurred outside of the shuttleboxes in a neutral context, followed by counterbalanced context freezing and cue freezing tests 1 day later. Since there were no differences in the pattern of lesion effects in Experiments 1 and 2, these data were combined into a single analysis. For the cue test, we used a two-way group (Sham vs. lesion) by TestPhase (Pre-CS vs. CS) ANOVA, treating TestPhase as a repeated measure. Rats in both groups showed little freezing pre-CS and strong freezing during the CS [Figure 6A; TestPhase: *F*_(1,33)_ = 258.5, *p* < 0.01]; however, MeA lesions did not significantly alter pre-CS or CS freezing [group × TestPhase: *F*_(1,33)_ = 1.2, *p* = 0.27]. For the context test, MeA-lesion rats froze less than Sham rats [Figure 6B; *t*_(33)_ = 2.29, *p* = 0.03].

### AA extinction

Since our PIT test involves AA extinction, we evaluated AA extinction directly in Sham and MeA-lesion rats. Rats were placed in the shuttleboxes and allowed to respond with shockers turned off for 60 min, then returned to the chambers 1 day later for the first aversive PIT test. PIT testing begins with AA extinction, thus, the first 5 min of the PIT test was used as the long-term memory test. Since there were no differences in the pattern of responding between Experiments 1 and 2, data from the two experiments were combined for analyses. Shuttling data are presented in Figure S4C in Supplementary Material in 5 min blocks. Within-session learning was assessed with a two-way group (Sham vs. lesion) × block (1–12) ANOVA, treating block as a repeated measure. Long-term memory was assessed by comparing shuttles during the last 5 min block of extinction acquisition (learning) with shuttles during the 5-min extinction test (memory) in a separate two-way group (Sham vs. lesion) × phase (learning vs. memory) ANOVA, treating phase as a repeated measure. Bonferonni post-tests were used to evaluate group differences during individual 5 min blocks. Sham and MeA-lesion rats shuttled equally during the first 5 min block of AA extinction [*t*_(396)_ = 0.37]. Shuttling decreased during the extinction session, and MeA-lesion rats extinguished slightly faster than Sham rats [group: *F*_(1,33)_ = 7.2, *p* = 0.01; block: *F*_(11,363)_ = 20.53, *p* < 0.01; group × block: *F*_(11,363)_ = 0.89, *p* = 0.55]. However, there was significant spontaneous recovery and both groups showed equivalent shuttling during the long-term memory test 1 day later [group: *F*_(1,33)_ = 1.97, *p* = 0.17; phase: *F*_(1,33)_ = 111.3, *p* < 0.01; group × phase: *F*_(1,33)_ = 0.02, *p* = 0.88].

### Aversive PIT

**Figure 7:**
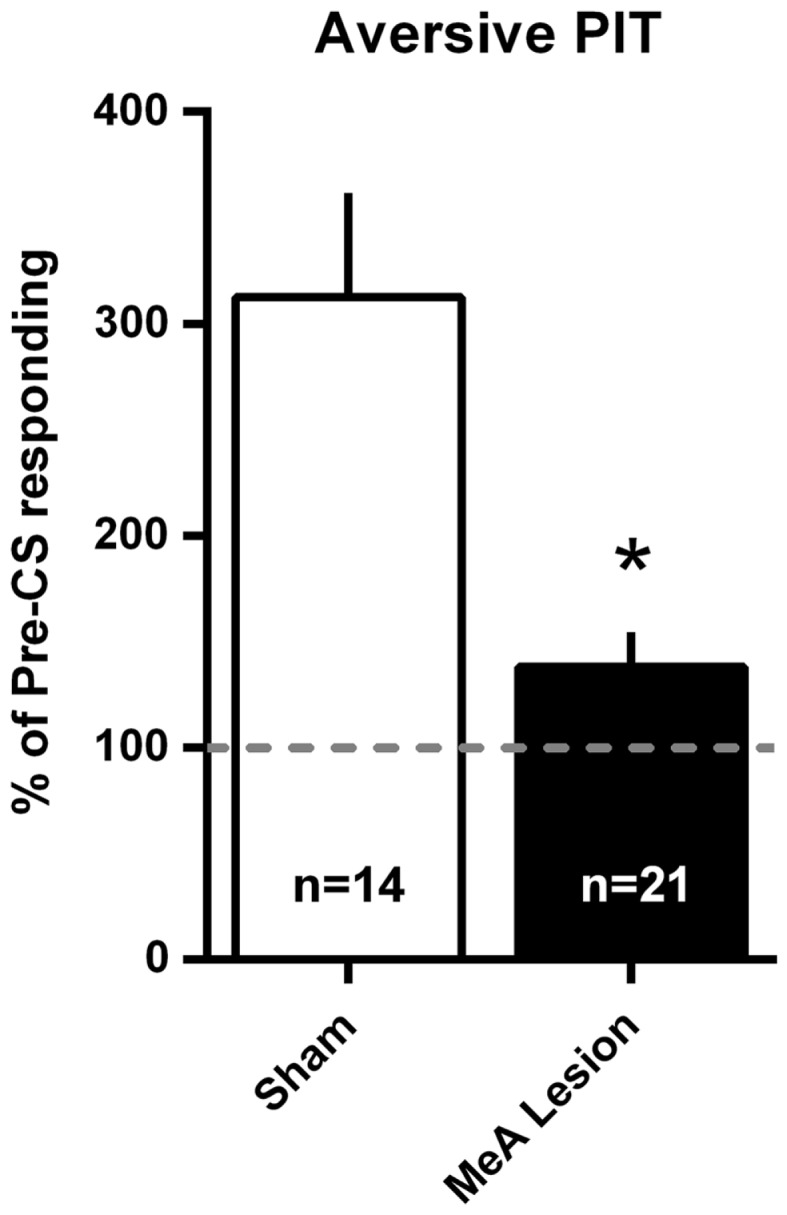
**MeA lesions block aversive PIT**. Rats received two PIT tests where a single CS presentation occurred after a baseline of AA responding in extinction. PIT is presented as the percent of pre-CS responding for the two sessions (see Material and Methods) for all MeA (filled bars, *n* = 21) and sham (open bars; *n* = 14) rats in Experiments 1 and 2. Dashed line represents the absence of PIT (pre- and post-CS AR rates were equal). *p < 0.05 vs. Sham controls.

Aversive PIT was evaluated by allowing rats to shuttle in extinction and comparing shuttling rate during the CS to the shuttling rate immediately preceding the CS. Since there were no differences in the pattern of responding between Experiments 1 and 2, data were combined into a single analysis. PIT data are presented as a percentage of pre-CS responding in Figure 7. For simplicity, and because there were no differences in PIT within the groups between tests, a mean PIT score was determined for each animal for the two PIT tests. Sham rats showed a significant increase in shuttling rate with the aversive CS presentation, and this PIT effect was absent in MeA-lesion rats [*t*_(33)_ = 3.915, *p* < 0.01].

### Shock reactivity

To ensure that MeA lesions do not affect US (footshock) reactivity, a separate group of rats received Sham (*n* = 8) or MeA-lesion (*n* = 6) surgery prior to a pain threshold test. Rats received footshocks in ascending intensity steps of 0.1 mA and the thresholds to flinch, audibly vocalize, or jump were recorded for each rat. Data were analyzed with a two-way group (Sham vs. lesion) × threshold (flinch, vocalize, jump) ANOVA, treating threshold as a repeated measure. There were increasing thresholds for eliciting flinch, vocalization, and jump responses; however, no differences in shock reactivity were observed between the groups [Figure S4B in Supplementary Material; group: *F*_(1,12)_ = 0.67, *p* = 0.43; threshold: *F*_(2,24)_ = 46.94, *p* < 0.01; group × threshold: *F*_(2,24)_ = 0.45, *p* = 0.65].

## Discussion

The present experiments expand our understanding of aversive conditioned motivation and provide novel information regarding the role of MeA in generating defensive responses. Our major novel results are: (1) MeA lesions abolish aversive PIT without affecting Pavlovian freezing to the PIT CS or baseline AA behavior, and (2) MeA lesions impair USV and freezing reactions to contextual threats. Control experiments and secondary analyses suggest that these effects are not explained by differences in locomotor activity, shock reactivity, total shocks received, or AA extinction. We also confirmed a role for MeA in predator odor avoidance. Together, these data suggest that MeA processes uncertain threats and may motivate ARs through activation of a general arousal-like state. These points are discussed in more detail below.

### Selectivity of MeA lesions

Electrolytic lesions were created by passing current through a monopolar electrode tip at four MeA sites per hemisphere. Histology revealed significant bilateral damage to MeA that completely spared damage to LA and BA, and largely spared damage to adjacent CeA and accessory basal nucleus. The cortical nucleus was moderately damaged in some animals, and the optic tract medial to MeA was damaged in nearly all cases. Damage to the optic tract may have affected vision in MeA-lesion animals; however, unsignaled AA depends critically on feedback stimuli (Bolles and Popp, 1964), which were visual in our paradigm, and rats with MeA lesions had no impairment in AA learning or performance. Visual cues are likely important for contextual conditioning, thus context data should be interpreted with caution. With MeA lesions, there is also some concern that amygdalofugal fibers running between MeA and CeA are damaged. However, others have reported that simultaneous bilateral lesions of the amygdalofugal pathway lead to aphagia, adipsia, and death (Liang et al., 1990). Although we recorded no mortality post-surgery for our Sham rats, 12 rats died post-surgery in the MeA-lesion group. Thus, we suspect that MeA lesions that significantly damaged the amygdalofugal pathway lead to premature death and assume that our final MeA-lesion groups had minimal damage to this pathway.

### MeA is not required for active avoidance

To our knowledge, MeA function has never been evaluated with AA paradigms. The present experiments were largely inspired by results from a recent c-Fos analysis following training with an identical unsignaled AA protocol (Martinez et al., 2013). That study, which focused on individual differences in AA behavior and competing Pavlovian reactions, found greater MeA c-Fos activation after an AA test in good vs. poor avoiders. This led us to hypothesize that MeA is required for AA performance. However, in the present studies, we found no effects on ARs or ERs with pre- or post-training lesions. This strongly suggests that MeA is not required for the reinforcement or motivation of AA responding. Since c-Fos studies are only correlational, it is quite possible that differences in MeA c-Fos simply reflect differences in afferent regions that directly mediate AA behavior. Indeed, in our previous study, we also found that AA behavior correlated with c-Fos in LA, BA, CeA, and IL-PFC. Each of these regions has been implicated in AA performance with loss of function studies (Poremba and Gabriel, 1997, 1999; Choi et al., 2010; Lazaro-Munoz et al., 2010; Moscarello and LeDoux, 2013) and each sends projections to MeA (Hurley et al., 1991; Pitkänen et al., 1997; Pitkänen, 2000).

### Role of MeA in Pavlovian defensive reactions

Our present data suggest that MeA is not required for the learning or expression of conditioned freezing to a discrete auditory cue. However, in several experiments, we found impairments in conditioned freezing to contextual cues, both in the AA context, and in a second conditioning context where the AR was not available. This was true even in our post-training lesion experiment with good avoiders, where total shock levels did not differ between groups (Figure 5B). It is notable that MeA lesions did not completely block context freezing, and in experiment 1, when we controlled for total shocks, MeA lesions did not significantly impair freezing in the AA context (Figure S2 in Supplementary Material). Thus, the results suggest that MeA plays a peripheral, not essential, role in Pavlovian context freezing.

MeA has received some attention in Pavlovian threat conditioning studies. Two studies, using a conditioning procedure similar to ours, found that pre-training MeA lesions failed to affect freezing to a tone CS previously paired with footshock (Nader et al., 2001; Holahan and White, 2002). However, another study found that inactivation of MeA blocks the expression of fear-potentiated startle to olfactory, visual, and contextual cues (Walker et al., 2005). A fourth study found that post-conditioning lesions of MeA had no effect on context freezing, but did block context-elicited neuroendocrine responses (Yoshida et al., 2014). The notion that MeA participates in contextual threat reactions appears to be supported by studies of neural activity in rats (Knapska et al., 2007; Trogrlic et al., 2011) and humans (Alvarez et al., 2008). Together, these findings suggest that MeA at least modulates contextual threat reactions, but has little role in Pavlovian reactions to discrete threat cues. This interpretation seems consistent with a role for MeA in extended amygdala processing of uncertain threats (Sullivan et al., 2004), defined as threats that are weakly correlated with the US, threats that lack temporal precision, or threats unlinked to any particular AR (Seligman et al., 1971; Rosen and Donley, 2006; Rau and Fanselow, 2007; Davis et al., 2010).

We also found that MeA lesions severely impaired USV responding in the AA context, even when the number of shocks was similar between MeA-lesion and Sham groups. We are aware of no studies that evaluated the role of MeA in conditioned USV reactions, however, USVs have been observed with stimulation of the basolateral amygdala complex (BLA: LA + BA) and periacqueductal gray (PAG; Kim et al., 2013). Lesion studies suggest that BLA is necessary for conditioned USVs (Koo et al., 2004). Interestingly, this same study found that electrolytic lesions of CeA impaired USVs, but excitotoxic lesions had only a modest effect. The authors suggest that BLA fibers passing through CeA to some unknown effector region are important for conditioned USVs. Since MeA receives inputs from BLA and projects to PAG (Canteras et al., 1995; Pitkänen et al., 1997), our data raise the interesting possibility that MeA links contextual threat representations to USV effector regions. Our data also suggest that MeA-mediated defensive reactions, like USVs, are not incompatible or directly competing with active ARs; MeA-lesion rats emitted comparatively few USVs, but were no better at acquiring or performing the AR. This contrasts with CeA-mediated reactions like freezing, which constrain AA performance (Lazaro-Munoz et al., 2010; Moscarello and LeDoux, 2013).

Lastly, our data are not inconsistent with studies showing that conditioned freezing and USVs are correlated, and proportional to anxiety states in rats (e.g., Borta et al., 2006). Defensive responses are believed to be arranged hierarchically, and are often mediated by different brain regions. However, these brain regions are components of larger survival circuits that produce coordinated and dynamic responses to threats (LeDoux, 2014). It is likely that factors responsible for trait anxiety influence multiple parts of the circuit and multiple defensive behaviors, especially in response to similar threats.

### MeA is necessary for PIT to a general threat cue

Pavlovian–instrumental transfer procedures have been widely employed in appetitive studies to elucidate the psychological and neural mechanisms of conditioned motivation. Although instrumental procedures themselves rely on conditioned motivation for response performance, they are not ideal for studying conditioned motivation because learning is gradual and it is difficult to differentiate between reinforcement and motivation processes. The PIT test is entirely performance-based and allows one to study Pavlovian motivation of instrumental actions in isolation (Estes, 1948; Lovibond, 1983).

Using our recently developed aversive PIT procedure (Campese et al., 2013), where Pavlovian threats facilitate unsignaled (Sidman) AA responding, we found that electrolytic lesions of LA or CeA blocked PIT, but lesions of BA did not (Campese et al., 2014). Importantly, in this experiment, unsignaled AA was overtrained, which leads to amygdala-independent AA performance (Poremba and Gabriel, 1999; Lazaro-Munoz et al., 2010). This allowed us to use lesions to evaluate PIT even though the same pre-training lesions normally impair AA acquisition (Lazaro-Munoz et al., 2010). In the present studies, we found that MeA lesions completely blocked aversive PIT, but had no effect on Pavlovian freezing to the CS or baseline instrumental avoidance. Thus, MeA is the first region to show a selective role in aversive transfer. LA is required for Pavlovian conditioning, AA, and PIT (Nader et al., 2001; Choi et al., 2010; Lazaro-Munoz et al., 2010; Campese et al., 2014). BA is required for AA and expression of Pavlovian conditioning (Anglada-Figueroa and Quirk, 2005; Choi et al., 2010; Lazaro-Munoz et al., 2010). And CeA is required for Pavlovian conditioning and PIT, but opposes AA expression (Nader et al., 2001; Choi et al., 2010; Lazaro-Munoz et al., 2010).

The present data may help refine our understanding of the complicated role that CeA plays in aversive conditioned motivation. It is unclear how CeA could mediate both Pavlovian reactions like freezing and facilitate instrumental actions like shuttling (PIT). CeA is known to mediate different response types via cell-type specific projections to different effector region (Huber et al., 2005; Viviani et al., 2011). CeA has also been shown to mediate both active and passive defensive responses (Gozzi et al., 2010), depending on local circuit activity and, perhaps, regulation by IL-PFC processes (Moscarello and LeDoux, 2013). Our results suggest alternative possibilities: (1) direct CeA projections could relay conditioned threat information to MeA even while outputs mediating Pavlovian freezing are inhibited, or (2) direct projections from LA to MeA could relay conditioned threat information necessary for PIT. Note that CeA has been implicated in aversive PIT only with electrolytic lesions that damage fibers of passage. Our USV data combined with previous findings (Koo et al., 2004; Kim et al., 2013) suggest that LA fibers coursing through CeA to MeA are important for conditioned USVs, and our MeA lesions impaired aversive PIT and conditioned USVs in the same animals, suggesting a common mechanism. These pathway specific hypotheses could be tested with disconnection lesions, inactivation of CeA, or more precise targeting of projections with optogenetic or chemogenetic techniques (Rogan and Roth, 2011; Aston-Jones and Deisseroth, 2013).

It is important to mention that appetitive PIT procedures have identified both outcome-specific and general forms of conditioned motivation. These complex procedures simultaneously evaluate multiple responses, CS and US combinations in the same animal during the same session (e.g., Corbit and Balleine, 2005). In brief, CSs selectively facilitate responses that are linked to the same US (specific PIT). Thus, when presented with a cue predicting sucrose, rats will selectively increase pressing on a bar that previously earned sucrose over a bar that earned food pellets. However, a CS linked to a third appetitive US (e.g., polycose) that was not available during bar-press training, will facilitate responding on both sucrose and food-pellet bars (general PIT). In appetitive studies, specific PIT depends on associations between the CS and specific sensory features of the US, and is BLA-dependent (Corbit and Balleine, 2005). General PIT depends on associations between the CS and “affective” properties of the US, and is CeA dependent (Hall et al., 2001; Holland and Gallagher, 2003; Corbit and Balleine, 2005). Thus in general PIT, CS presentations are assumed to activate a general arousal-like state that can motivate many instrumental responses linked to USs of the same valence. These complex procedures are more difficult to develop with aversive studies, however, there is reason to believe our procedure produces general PIT. First, although the reinforcement mechanism in AA is unknown, it is clearly different from the reinforcement in Pavlovian threat conditioning. In AA, learning occurs on trials where the US is omitted, whereas in threat conditioning, learning occurs on trials where the US is presented. This mismatch between reinforcers suggests that specific PIT is not possible with our procedure. Second, appetitive studies suggest that a response choice is necessary for specific PIT (Corbit and Balleine, 2005); even when USs match, PIT is CeA dependent when only one instrumental response is available, as in our procedure (Holland and Gallagher, 2003). Thus, we hypothesize that threats in our simple PIT procedure activate a general defensive state that can motivate any avoidance response available to the animal.

**Figure 8:**
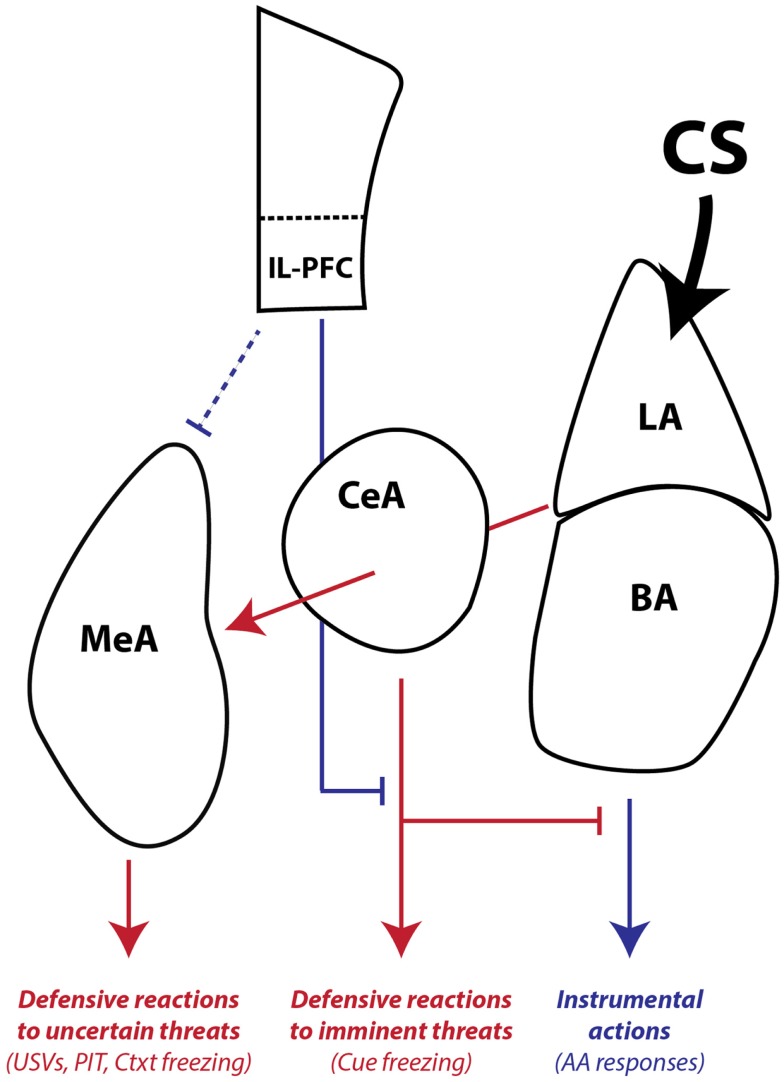
**Working model of amygdala pathways mediating defensive reactions, AA, and aversive PIT**. LA is primarily involved in learning and storing Pavlovian CS–US associations. Once the CS gains affective valence, it can be used by downstream areas to generate wide-ranging defensive behaviors. LA and BA are required for instrumental AA, whereas CeA and MeA are not. CeA is necessary for expressing Pavlovian reactions to imminent threats. MeA, as part of the medial extended amygdala, mediates defensive reactions to uncertain or distant threats and aversive PIT to general threats that are not part of the AA memory associative structure. IL-PFC can regulate amygdala-mediated defensive reactions and facilitate AA performance. Blue lines denote pathways that can promote instrumental action and block defensive motivational states. Red lines denote pathways that can promote defensive reactions and defensive motivational states. The line connecting IL-PFC to MeA is dashed because little is known about the influence of this pathway on defensive reactions and aversive PIT. The line connecting LA to MeA passes through CeA since it is not yet clear whether CeA is necessary for conditioned USVs and PIT or whether fibers passing through CeA relay critical information directly to MeA. LA, lateral amygdala, BA, basal amygdala, CeA, central amygdala, MeA, medial amygdala, IL-PFC, infralimbic prefrontal cortex, USV, 22 kHz ultrasonic vocalizations, PIT, Pavlovian-instrumental transfer, Ctxt, context, CS, conditioned stimulus.

Finally, our combined studies on AA and PIT suggest that there is another distinction in conditioned motivation mechanisms that relates to the role of the CS in the instrumental associative structure. Both AA and PIT rely on conditioned motivation mechanisms to generate AA responding, so why would they depend on such different neural pathways? Early in AA training, the CS (or warning signal) is transformed into a threat by pairing with the US. However, once the AR is learned, the CS functions as a discriminative, or occasion-setting, stimulus that signals when the instrumental contingency is in operation (Ross and LoLordo, 1987; Rescorla, 1990). In our PIT procedure, the PIT CS is never present during AA training and cannot be part of the instrumental memory structure. Thus, our data are consistent with a model where: (1) LA is necessary for threat learning, (2) BA is necessary for signaling when an AR is available to avoid a specific US, and (3) CeA and MeA are necessary for motivation of ARs when threats are uncertain or unlinked to available ARs, through activation of a central defensive state (Figure 8).

### Limitations

We chose to use electrolytic lesions to evaluate the role of MeA in learned and innate defensive responses. Electrolytic lesions are often preferred for initial investigations of the necessity of brain regions (Cain and LeDoux, 2008b). There are several reasons for this: (1) they can clearly rule out a necessary role for a brain region, since effective lesions leave no functional brain tissue, (2) compared to chemical lesions, inactivations, or techniques that depend on viral infection, it is easier to control the spatial extent of affected tissue, and (3) it is easy to confirm the manipulation with basic histological techniques. However, electrolytic lesions are permanent and damage fibers of passage (Kim et al., 2013), which can sometimes lead to misleading results if there are compensatory changes in the brain or if fibers of passage in a region, but not cell bodies, are necessary for a particular function. Electrolytic lesions may be most problematic for the interpretation of context freezing deficits, as the optic tract was clearly damaged in most animals. Although rodents likely use all sensory modalities in creating a representation of context, visual cues are clearly important, and these results should be interpreted with caution. Ultimately, it is important to confirm the effects of electrolytic lesions with techniques that are reversible and do not damage fibers of passage. Exciting new techniques also allow for control neural activity that is cell-type specific, reversible and even pathway specific (by controlling projections between brain regions) (Rogan and Roth, 2011; Aston-Jones and Deisseroth, 2013). We are currently pursuing such studies to confirm the roles of LA, BA, CeA, MeA, and IL-PFC in threat conditioning, AA and aversive PIT.

As mentioned above, our PIT procedure cannot differentiate between outcome-specific and general forms of conditioned motivation. Although we are developing procedures that may ultimately address these issues, these procedures are inherently more difficult to develop than appetitive PIT procedures. This is mainly because hungry rats are much more likely to behave actively when presented with multiple food options, whereas rats experiencing multiple threats and aversive USs tend to cease active behavior and freeze. It is important to point out that aversive PIT studies have lagged far behind appetitive PIT studies, and it will take time to develop the ideal procedures. However, our simple PIT procedure is already generating novel and important information about aversive conditioned motivation, as did the early appetitive PIT studies that also used simple procedures (Estes, 1948; Lovibond, 1983).

Lastly, our interpretation of the USV findings assume that these are conditioned reactions elicited by Pavlovian contextual cues. This is largely because prior studies interpret USVs this way (e.g., Koo et al., 2004), and because USVs were elicited in the shock-paired AA context, and post-shock responses like freezing are known to be conditioned, not unconditioned, reactions (Fanselow, 1986). However, it is possible that USVs represent unconditioned reactions to US presentation in the AA context. Others have reported 22 kHz USV responses to unconditioned threats, including predators (Blanchard et al., 1991), and direct stimulation of pathways believed to relay US information to the amygdala also trigger USVs (Kim et al., 2013). Further, it was not uncommon in our studies that rats began emitting USVs after receiving the first shock during AA training sessions (not upon entering the chamber). However, this alternate interpretation of USVs would not significantly change our conclusions and would only suggest that MeA has a dual role in processing conditioned threats and mediating unconditioned responses to naturally aversive stimuli. This seems likely anyway, given the clear role in aversive PIT and in defensive responses to predator odor cues (e.g., Rosen et al., 2008; Figure S3 in Supplementary Material).

## Conclusion and Clinical Implications

In conclusion, our studies reveal an essential and selective role for MeA in aversive PIT. They also suggest that MeA is critical for processing uncertain or general threats and generating lower-level “anxiety-like” defensive responses. Although we cannot know what the rat is feeling during these tasks (LeDoux, 2014), it is likely that these forms of threat processing relate to human anxiety disorders. Human anxiety is characterized by defensive reactions to often uncertain threats (Tolin et al., 2003), and AA mechanisms likely relate to both adaptive (LeDoux and Gorman, 2001) and maladaptive coping strategies (McGuire et al., 2012). Aversive PIT demonstrates how threat cues can invigorate, or re-invigorate, AA behavior, even after it is extinguished. In the case of adaptive ARs, PIT mechanisms could contribute to beneficial active coping strategies in resilient individuals. However, in the case of maladaptive ARs, PIT mechanism could trigger a relapse to pathological behavior even after seemingly successful treatment. Several recent reports demonstrate that aversive PIT occurs in humans and may depend on similar neural pathways (Nadler et al., 2011; Geurts et al., 2013; Lewis et al., 2013). These studies, along with mechanistic studies in rodents, hold promise for discovering novel and improved treatments for human anxiety disorders characterized by impaired or inappropriate avoidance responding.

## Author Contributions

Margaret Grace McCue and Dr. Christopher K. Cain designed the experiments. Ms. Margaret Grace McCue conducted all of the surgeries, behavioral testing and histology for lesion verification. Ms. Margaret Grace McCue, Dr. Christopher K. Cain, and Dr. Joseph E. LeDoux analyzed the results, wrote the manuscript and approved the final manuscript for submission.

## Conflict of Interest Statement

The authors declare that the research was conducted in the absence of any commercial or financial relationships that could be construed as a potential conflict of interest.

## Supplementary Material

The Supplementary Material for this article can be found online at http://www.frontiersin.org/Journal/10.3389/fnbeh.2014.00329/abstract
