# Supplementary material for: Medial Amygdala Lesions Selectively Block Aversive Pavlovian–Instrumental Transfer in Rats
Source: Front Behav Neurosci. 2014 Sep 18;8:329. doi: 10.3389/fnbeh.2014.00329 (PMC4166994; doi:10.3389/fnbeh.2014.00329)
Supplement: Supplementary file 1 [file Presentation1.PDF]

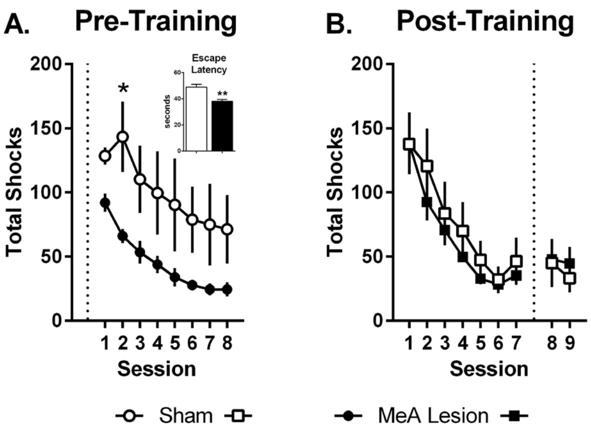

Supplemental Figure 1. Pre-training MeA lesions reduce total shocks during AA training. (A) Total number of shocks per session during AA training for rats with pre-training MeA (filled circles/bar; n=13) or sham (open circles/bar; n=9) lesions. *Inset*: latency to emit an escape shuttle following a shock presentation during Session 1. Note that latencies greater than 5s indicate that multiple shocks were delivered before an escape shuttle was observed. MeA-lesion rats escaped sooner than Sham rats leading to less total shocks. **(B)** Total number of shocks per session during AA training (sessions 1-7) and AA tests (sessions 8-9) for rats with post-training MeA (filled squares; n=8) or sham (open squares; n=5) lesions. *p<0.05 vs. Sham controls

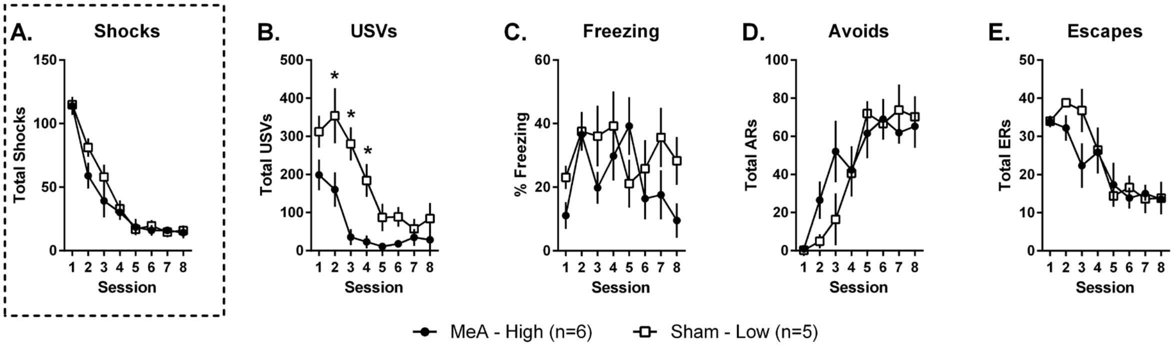

Supplemental Figure 2. MeA lesions impair USVs even when total shocks are equal. Rats in experiment 1 (pre-training lesions) were divided by total shocks received (dotted box) during session 1. Here we compare all AA training behaviors in rats from the top half of the MeA lesion group (filled circles) and the bottom half of the sham group (open squares). **(A)** total number of shocks per session during AA training. **(B)** total number of USVs per session. **(C)** percent freezing during first 2-minutes of each session. **(D)** total number of AA responses (ARs) per session. **(E)** total number of escape responses (ERs) per session. *$p<0.05$ vs. Sham controls

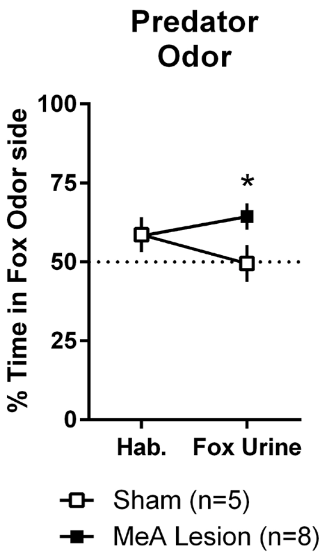

Supplemental Figure 3. MeA lesions reduce predator odor avoidance. Percent time spent in predator odor chamber (above dotted line) and adjacent control chamber (below dotted line) during habituation sessions and test sessions with fox urine odor. *$p<0.05$ vs. Sham controls

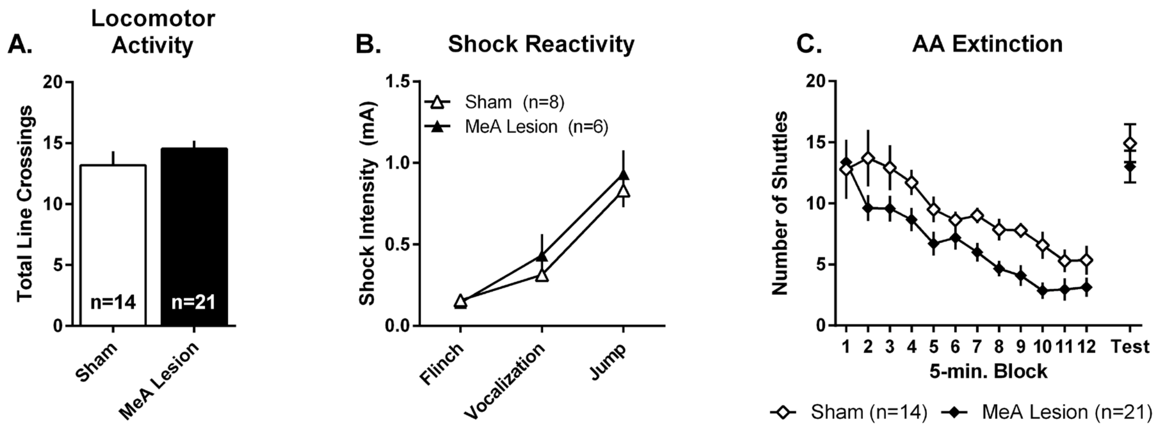

Supplemental Figure 4. MeA lesions do not impair locomotor activity, shock reactivity, or AA extinction memory. (A) total number of line crossings during habituation sessions to the predator odor test context for all MeA and Sham lesion rats in experiments 1 and 2. **(B)** Threshold to flinch, vocalize and jump to brief footshocks of ascending intensity (in mA) for rats in experiment 3. **(C)** within-session AA extinction displayed as total number of shuttles per 5-minute block (1-12), and long-term AA extinction assessed 1d later during the first 5-minutes of the PIT test session (Test) for all rats in experiments 1 and 2. *p<0.05 vs. Sham controls
